# Chromatographic and Thermal Characteristics, and Hydrolytic and Oxidative Stability of Commercial Pomegranate Seed Oil

**DOI:** 10.3390/foods13091370

**Published:** 2024-04-29

**Authors:** Marta Siol, Agnieszka Dudek, Joanna Bryś, Diana Mańko-Jurkowska, Eliza Gruczyńska-Sękowska, Sina Makouie, Bharani Kumar Palani, Marko Obranović, Piotr Koczoń

**Affiliations:** 1 Department of Chemistry, Institute of Food Sciences, Warsaw University of Life Sciences, Nowoursynowska St. 159c, 02-787 Warsaw, Poland; marta_siol@sggw.edu.pl (M.S.); romaniuk.agnieszka11@gmail.com (A.D.); joanna_brys@sggw.edu.pl (J.B.); diana_manko-jurkowska@sggw.edu.pl (D.M.-J.); eliza_gruczynska@sggw.edu.pl (E.G.-S.); sina_makouei@sggw.edu.pl (S.M.); bharani_palani@sggw.edu.pl (B.K.P.); 2 Department of Food Engineering, Faculty of Food Technology and Biotechnology, University of Zagreb, Pierottijeva 6, 10000 Zagreb, Croatia; mobran@pbf.hr

**Keywords:** pomegranate seed oil, fatty acid composition, fatty acid distribution, oxidative stability

## Abstract

The current investigations were aimed at the determination of the hydrolytic and oxidative stability of commercial pomegranate seed oils provided by four different producers, and to assess the oils’ primary quality parameters. During storage, many changes occur in oils that can significantly affect their quality. The oils were tested for acid and peroxide values, fatty acid profile, and their distribution between the *sn*-1,3 and *sn*-2 positions of triacylglycerols. The oxidative stability was also determined, and melting curves were plotted for the oils. The analyzed oils were stored for one month in a dark place at refrigerator temperature. Based on the obtained results, it was found that the acid values for most oils did not exceed the permissible level determined by the Codex Alimentarius. However, in all oils, the peroxide value exceeded the permissible level set by the standard EN ISO 3960:2017-03 and the Codex Alimentarius after the one-month storage period. The examined pomegranate seed oils were found to be valuable sources of polyunsaturated fatty acids, especially punicic acid, which was the most abundant fatty acid present in these oils. In all analyzed oils, linoleic acid predominated in the *sn*-2 position of the triacylglycerols. Pomegranate seed oils did not exhibit good oxidative stability, as the oxidation induction times for all tested oils were very short. The storage period significantly affected the content of the primary oxidation products and oxidative stability of the oils.

## 1. Introduction

The pomegranate (*Punica granatum* L.), belonging to the family Punicaceae, has been consumed in various cultures for thousands of years. Pomegranate fruits were widely used in ancient times, symbolizing fertility, abundance, and immortality [1,2]. They were used in the treatment of ulcers, diarrhea, hemorrhages, microbial infections, and respiratory system disorders [3,4]. Pomegranate fruits are excellent source of several minerals, e.g., phosphorus, magnesium, silicon, potassium, calcium, and iodine. They also provide consumers great amounts of A, C, B, and E vitamins, as well as organic acids and phenolic compounds [5,6]. The food industry utilizes these fruits in huge amounts to produce juices, jellies, jams, wines, and flavoring and coloring for beverages [2]. Pomegranates are abundant in seeds, which are usually removed during processing and wasted, although the literature suggests that these by-products can be a rich source of biologically active compounds and nutrients that can be isolated and utilized, aligning with the EU’s closed-loop policy, the goals of sustainable development policy, as well as the “zero” or “less” waste concept [7]. Pomegranate seeds are particularly interesting by-products due to the properties of the oil they contain.

The content of oil in pomegranate seeds ranges from 12 to 20% on a dry-weight basis. Further, the content of conjugated fatty acids in pomegranate seed oil ranges from 65 to 80% [8]. Unsaturated fatty acids are contained in pomegranate seed oil, as well as tocopherols, which exist mainly in the form of both α and γ-tocopherol. β-campesterol, sitosterol, and stigmasterol represent phytosterols present in the oil of pomegranate seeds [9]. The presence of significant amounts of conjugated linolenic acid that is punicic acid is an extraordinary and characteristic feature of pomegranate oil. Punicic acid, which exhibits, among others, antidiabetic, antiatherogenic, antiproliferative, and anticancer properties, is beneficial for human health [10]. In particular, it can assist with the treatment of breast cancer and prostate cancer via the proliferation of specific cancer calls [11,12]. Furthermore, it shows strong anti-inflammatory and anti-edema properties, as well as high antioxidant activity [13,14]. Studies conducted with rats have shown that supplementation with pomegranate seed oil leads to the accumulation of fatty mass, hence causing them to gain weight. This way, obesity and insulin resistance (usually diet-induced) can be combated or at least reduced [15]. Additionally, research conducted by Khajebishaka et al. [16] indicated the potential of punicic acid in the treatment of type 2 diabetes.

Vegetable oils are subject to an oxidation process due to the high content of unsaturated fatty acids. Lipid oxidation depends on many factors, including fatty acid composition, endogenous antioxidants, catalysts, as well as primary and secondary oxidation products, especially in the case of cold-pressed oils. Oils obtained by cold-pressing are characterized by higher initial acid and peroxide values, indicating their low oxidative and hydrolytic stability. To obtain oils with better stability, they undergo a refining process. During this process, free fatty acids and primary oxidation products are removed, resulting in refined oils with improved stability and longer shelf life [17,18]. The use of damaged seeds when pressing oil may also reduce their oxidative stability and increase their acid and peroxide values. In addition, the stability of the oil is influenced by roasting the seeds and the pressing temperature.

Oxidative stability is one of the most important indicators of the quality and shelf life of edible oils. Several methods for assessing oxidative stability have been developed, including accelerated methods based on measuring oil oxidation induction times. The Rancimat test and pressure differential scanning calorimetry (PDSC) are commonly used due to their simplicity and quickness, and the reproducibility of the results. PDSC is capable of representing field performance within a short testing time, does not require chemical reagents, and only requires a few milligrams of sample for analysis [19]. Additionally, elevated pressure reduces sample volatility, thereby raising boiling points and enhancing the concentration of reacting gases. Consequently, this facilitates the use of lower test temperatures or shorter test times at the same temperatures [20].

This study aimed to evaluate the quality of commercially available pomegranate seed oils immediately after opening and after a one-month storage period. Parameters such as the peroxide and acid values, composition and distribution of fatty acids in the structure of triacylglycerols (TAGs), oxidative and hydrolytic stability, and melting characteristics were considered. The appropriate instrumental analytical methods, e.g., automatic titration, chromatography, enzymatic hydrolysis, pressure differential scanning calorimetry, and differential scanning calorimetry (DSC), were conducted. Every applied method was dedicated to determining significant features that indicate oil quality. Additionally, health indices of atherogenicity, thrombogenicity, and the ratio of hypocholesterolemia to hypercholesterolemia were examined to inform about the potential influence of the fatty acids present in pomegranate seed oil on human health. Many complimentary instrumental methods have been applied to collect appropriate data to characterize pomegranate seed oils from different points of view, that is, physical–chemical and nutritional–healthy oil properties.

## 2. Materials and Methods

### 2.1. Materials

Four cold-pressed, unrefined pomegranate seed oils were used as research material. The oils were purchased in Poland; however, the country of origin of all four products was Turkey. The conditions of their production and transport were unknown. Based on the manufacturers’ instructions, the oils were cold-pressed and were 100% natural without additional antioxidants. When selecting the oils, their expiration date was taken into account. The oils were tested immediately after opening (4 months before the expiration date), and after one month of refrigerated storage in the original dark glass bottles. For each analysis, samples of all tested oils were taken from the original bottles.

### 2.2. Methods

#### 2.2.1. Determination of Acid and Peroxide Values

The degree of hydrolysis of the investigated oils was determined by the acid value according to the AOCS method (AOCS Official Method Te 1a-64) [21]. The content of primary oxidation products of the oils was examined based on the peroxide content as per the AOCS method (AOCS Cd 8b-90) [22]. The acid and peroxide values were determined immediately after opening and after one month of storage.

#### 2.2.2. Determination of Fatty Acid Composition by Gas Chromatography

The following standards: EN ISO 5509:2001 and EN ISO 5508:1996 [23] were applied to determine the composition of fatty acid present in the oils. A gas chromatograph YL6100 GC (Young Lin Bldg., Anyang, Hogyedong, Republic of) equipped with a BPX-70 capillary column (60 m length, 0.25 μm film thickness, and 0.25 mm internal diameter) as well as a flame ionization detector (FID) was used to analyze the oils. The gas to carry gaseous methyl esters inside the column was nitrogen. The initial temperature was 70 °C for 0.5 min, while the final one was 225 °C. The temperature change was 15 °C/minute, 1.1 °C/minute, and 30 °C/minute in the temperature ranges of 70–160 °C, 160–200 °C, and 200–225 °C, respectively. The temperature was kept constant for 12 min when it reached 200 °C. The detector and injector temperatures were 250 °C and 225 °C, respectively. The abundance percentage of each fatty acid was determined [24]. 

#### 2.2.3. Distribution of Fatty Acids in Triacylglycerols Using Enzymatic Hydrolysis

The tested oils were subjected to enzymatic hydrolysis using pancreatic lipase specific for breaking the ester bonds in the *sn*-1,3 position. Hydrolysis was performed in a thermostated laboratory shaker for 20 min at 40 °C. To a flask containing 400 mg of oil 8 cm^3^ of an aqueous solution of TRIS at pH 8.0 and a concentration of 1 mol/dm^3^ were added, as well as 0.5 cm^3^ of a 2.2% solution of CaCl_2_ and a solution of 200 mg of pancreatic lipase dissolved in 0.2 cm^3^ of a 1% aqueous solution of bile acid salts. The reaction was stopped by adding 15 cm^3^ of diethyl ether and 5 cm^3^ of 6 mol/dm^3^ hydrochloric acid. Enzymatic hydrolysis products dissolved in diethyl ether were separated using the preparative thin-layer chromatography (TLC) technique. For this purpose, 20 × 20 cm plates covered with silica gel were used. A chromatogram was developed using a mixture of 50 cm^3^ hexane, 50 cm^3^ diethyl ether, and 1 cm^3^ acetic acid. The silica gel, along with isolated *sn*-2 monoacylglycerols, was removed from the plates [25]. The fatty acid composition of the obtained *sn*-2 monoacylglycerols was determined by gas chromatography [24]. Based on the compositions of the isolated *sn*-2 monoacylglycerols and the starting triacylglycerols, the composition of the fatty acid in the *sn*-1,3 positions was determined. The following equations were used:

(1) $$sn-1,3=\frac{3\times ({FA}_{in\;TAG})-({FA}_{in\;sn-2\;MAG})}{2}$$

(2) $$sn-2=\frac{({FA}_{in\;sn-2\;MAG})\times 100\%}{3\times \left({FA}_{in\;TAG}\right)}$$

where:

*sn*-1,3—content of a given fatty acid share in the *sn*-1 and *sn*-3 positions of TAG [%];

*sn*-2—content of a given fatty acid share in the *sn*-2 positions of TAG [%];

FA_in TAG_—content of a given fatty acid in the starting triacylglicerols (TAGs) [%];

FA_in_ *_sn_*_-2 MAG_—content of a given fatty acid in *sn*-2 monoacylglycerols (MAGs) [%].

#### 2.2.4. Health Indicators of Oils

The fatty acid profile was used to calculate the health indicators. The atherogenicity index (AI) and the thrombogenicity index (TI) were obtained from Equations (3) and (4), respectively [26]; the hypocholesterolemia/hypercholesterolemia (h/H) ratio was obtained from Equation (5) [27]:

(3) $$AI=\frac{C12:0+\left(4\times C14:0\right)+C16:0}{\sum PUFA}$$

(4) $$TI=\frac{C14:0+C16:0+C18:0}{0.5\times \sum MUFA+\left(0.5\times \sum n-6PUFA\right)+\left(3\times \sum n-3PUFA\right)+\frac{n-3}{n-6}}$$

(5) $$h/H=\frac{cis-C18:1+\sum PUFA}{C12:0+C14:0+C16:0}$$

#### 2.2.5. Determination of Oxidative Stability Using the PDSC Method

Pressure differential scanning calorimetry using PDSC (DSC Q20 TA Instruments, Newcastle, WA, USA) was employed to determine the induction time for the oxidation reaction of oil. The weight of the oils used in the test ranged from 3 to 4 mg. Fat placed in an aluminum pan was oxidized at a pressure ranging from 1350 to 1400 kPa [28]. The samples were oxidized at 120 °C.

#### 2.2.6. Determination of Melting Characteristics Using the DSC Method

Melting characteristics were determined by differential scanning calorimetry using a DSC Q200 (DSC Q200 TA Instruments, Newcastle, WA, USA). All measurements were carried out in an atmosphere of nitrogen as a cooling medium, and the reference sample was an empty aluminum pan that was hermetically sealed. The mass of samples used for the test ranged from 3 to 4 mg. The analysis began by cooling the samples to –80 °C and then heating them at a rate of 5 °C/min to a final temperature of 80 °C.

#### 2.2.7. Statistical Analysis

Statistica 13 statistical software was used to consider the results obtained statistically. Analysis of variance (ANOVA) and Tukey’s test at a significance level of α = 0.05 were applied. Microsoft Excel 2010 was used to calculate the mean with standard deviation. All analyses were performed in triplicate.

## 3. Results and Discussion

### 3.1. Acid and Peroxide Values

The acid value is defined as milligrams of KOH needed to neutralize the free fatty acids present in 1 g of fat. As stated in the Codex Alimentarius [29], the acid value should not be greater than 4 mg KOH/g fat in cold-pressed oils. The peroxide value is a measure of peroxide content and determines the degree of oxidation, or rancidity, of fat. The peroxide value is limited for cold-pressed oils; according to EN ISO 3960:2017-03 [30], it should not exceed 10 meq O_2_/kg or, per the Codex Alimentarius [29], 15 meq O_2_/kg. Table 1 shows the changes in the average acid and peroxide values of the oils immediately after opening and after one month of storage.

The results show that pomegranate seed oil D was characterized by the highest acid value both at the beginning and at the end of storage. It was also the only tested oil that exceeded the recommended acid value standard for cold-pressed oils. In contrast, oils A and C were characterized by the lowest acid values, even after one month of storage. The initial acid values for pomegranate seed oils A and C were 1.97 and 1.76 mg KOH/g fat, respectively. The initial acid value of oil D was as high as 6.12 mg KOH/g fat. It was observed that after one month of storage, the acid values for all oils slightly increased. The statistical analysis carried out showed that the acid values determined immediately after opening were not statistically significantly different from the acid values after one month of storage for all the oils.

The lowest peroxide value immediately after opening was found for oil B, which was the only case where the peroxide value fulfilled the limits of the EN ISO 3960:2017-03 standard [30], amounting to 6.34 meq O_2_/kg fat. Oils A and C exceeded the limit of 10 meq O_2_/kg fat indicated in EN ISO 3960:2017-03 [30] but were below the limit according to the Codex Alimentarius [29]. However, the peroxide value of oil D was as high as 19.31 meq O_2_/kg fat and exceeded both permissible peroxide values set for cold-pressed oils. After a one-month storage period, a significant increase in primary oxidation products was observed in the tested pomegranate seed oils. The peroxide values for all the analyzed oils exceeded the limits set in both EN ISO 3960:2017-03 [30] and the Codex Alimentarius [29]. The peroxide values of the tested oils determined immediately after opening and after one month of storage were not statistically significantly different from each other.

In a study on pomegranate seed oil published by Drinić et al. [31], the acid value in fresh oil ranged from 0.74 to 0.91 mg KOH/g fat and did not differ significantly after a 12-day storage period. However, the peroxide value ranged from 0.52 to 5.75 meq O_2_/kg fat and almost doubled during storage. Bialek et al. [32] analyzed a set of eight pomegranate seed oil samples from online stores in China and Poland. The fresh oils just after opening were investigated. The acid values in the tested oils ranged from 0.06 mg KOH/g fat to 6.94 mg KOH/g fat, and the peroxide values ranged from 2.97 meq O_2_/kg fat to 13.9 meq O_2_/kg fat. Costa et al. [33] studied three pomegranate seed oils originating from Israel and Turkey, and their acid values were located in the range of 1.80–4.38 mg KOH/g fat, while the peroxide values were located in the range of 0.91–2.69 meq O_2_/kg fat. Comparing the results obtained in this study to those from other researchers, it can be concluded that the oils immediately after pressing were characterized by lower acid and peroxide values than commercial oils. The researchers showed significant differences in acid value among the commercial oils studied. In most of the other studies, the acid number of the oils before and after storage did not change significantly, whereas the peroxide number did—sometimes even doubling, exactly as for the oils investigated in this study.

It should be taken into account that the tested oils were commercial products. We do not know the quality of the seeds used for pressing or their humidity, damage, or storage conditions. Quality control of the raw material is necessary because the quality of the oil cannot be improved from unsuitable seeds [34]. Additionally, using damaged seeds for oil pressing may increase the acid and peroxide content. A high peroxide content may therefore indicate a significant degree of oxidation of individual oils before testing. 

### 3.2. Fatty Acid Composition

Pomegranate seeds, which account for 12–20% of the fruit’s total weight, are a rich source of lipids. Pomegranate seed oil is characterized by a high content of conjugated linolenic acids (CLnA), or isomers of linolenic acid, with three conjugated double bonds, usually at positions 9, 11, and 13 or 8, 10, and 12, with various combinations of geometric configurations, *cis* or *trans*. Pomegranate oil is characterized by a unique fatty acid profile, being a concentrated source of the CLnA isomer punicic acid. Punicic acid is the most important fatty acid of pressed pomegranate seed oil, accounting for 55–81% of all fatty acids. The main remaining fatty acids in terms of quantity are linoleic acid, oleic acid, palmitic acid, and stearic acid [33,35,36]. The fatty acid composition of the four pomegranate seed oils was determined using the gas chromatography method. The results are presented in Table 2.

Regardless of the storage period, C18:3 punicic acid (9c, 11t, 13c) accounted for the largest share in the three oils tested, while polyunsaturated fatty acids (PUFAs) accounted for the largest share in one oil. All the tested oils were rich sources of PUFAs and monounsaturated fatty acids (MUFAs), while saturated fatty acids (SFAs) and other fatty acids accounted for the smallest share. The average content of MUFAs in the tested pomegranate seed oils immediately after opening and after one month of storage was in the range of 7.77–20.51%. The monoenoic acids in the pomegranate seed oil consisted mainly of oleic acid (C18:1 n-9), and only a small amount was gondinic acid (C20:1 n-9). Oils A and C had the highest MUFA content. Immediately after opening, they contained 19.50% and 20.51% MUFAs, respectively. Oil D was characterized by a slightly lower MUFA content. Oil B had the lowest MUFA content, at 8.45%. After one month of storage, the proportion of MUFAs in pomegranate seed oils decreased. No statistically significant differences were observed in MUFA content for oils immediately after opening or after one month of storage, whereas significant differences were observed among oils from different manufacturers.

Linoleic acid (C18:2 n-6), α-linolenic acid (C18:3 n-3), and eicosatrienoic acid (C20:3 n-3) were the PUFAs contained in pomegranate seed oil, with linoleic acid accounting for the largest share in this group. After analyzing the content of PUFAs in the pomegranate seed oils tested immediately after opening, it was concluded that the highest content was in oil D, which contained 32.67% of them. For oils A and C, the average content of PUFAs was 28.10% and 29.46%, respectively. The lowest amount of PUFAs, 8.51% at the beginning of storage, was found in oil B, resulting from exceptionally low α-linolenic acid content. After one month of storage, it was observed that the proportion of PUFAs in oil D decreased slightly and amounted to 31.80%. Slight differences in PUFA content after one month of storage were also observed in the other oils, for which the average PUFA content was 27.92% in oil A, 7.29% in oil B, and 29.10% in oil C. A reduction in PUFA content after one month of storage was observed in all oils, but these differences were not statistically significant.

SFAs in pomegranate seed oils ranged from 6.40 to 8.11% and included palmitic acid (C16:0) and stearic acid (C18:0), as well as traces of arachidic acid (C20:0). Immediately after opening, the average SFA content was lowest in oils A and B and amounted to 7.80% and 7.25%, respectively. Oils C and D contained slightly more SFAs, with their average content reaching 8.05% and 8.11%, respectively. After one month of storage, the average SFA content decreased slightly in all oils.

Conjugated octadecatrienoic fatty acids constituted approximately 80% of the total content of fatty acids in pomegranate seed oil, especially punicic acid (with the following positions of double bonds in the carbon chain: C18:3 9c, 11t, 13c). Other isomers of conjugated linolenic acid (CLnA) found in pomegranate oil were catalpic (C18:3 9t, 11tr, 13c) and α-eleostearic (C18:3 9c, 11t, 13t) acids [9]. Among all pomegranate seed oils immediately after opening, the highest percentage content of punicic acid was found in oil B, which contained 67.36% of this fatty acid. A punicic acid content that was more than twice as low as that of oil B was identified in oil D. The differences in punicic acid content among the commercial oils were surprisingly large. An increase in CLnA content was observed in all the oils during storage. 

The fatty acid profile of oil B, with the highest content of punicic acid and the lowest content of linoleic acid, was similar to the profiles found in the literature for pure pomegranate seed oil [8]. It may mean that this oil does not contain any additives. Oil D had the lowest content of punicic acid and the highest content of linoleic acids, which may suggest the presence of another oil. Oils A and C were similar in terms of fatty acid content to oil D, which may mean that a different oil could have been added to them. Pomegranate seed oil was most likely mixed with another oil rich in linoleic acid. Such high contents of fatty acid can be found in sunflower, soybean, corn, and sesame oil [37]. According to Uncu et al. [38], these characteristic differences in fatty acid composition could be used as markers in the adulteration detection of pomegranate seed oils. 

Punicic acid was also identified as a major fatty acid in pomegranate seed oils by Dadashi et al. [39], with its percentage reaching 72.07–73.31%. There were slight differences in the content of individual fatty acids among the oils tested. The content of SFAs among the four tested varieties ranged from 7.45% to 8.1% and included palmitic, stearic, and arachidic acids. Among all tested varieties, the main MUFA was oleic acid, and the amount ranged from 8.31% to 9.77%. Eicosenoic acid (C20:1) as a MUFA was also identified in small quantities; the amount ranged from 0.90 to 1.08%. Linoleic acid (C18:2) levels ranged from 8.41% to 9.38%. α-Linolenic acid (C18:3) was also present in the oils studied, but in much lower amounts of less than 0.1%.

According to the study by Costa et al. [33] on a cold-pressed pomegranate seed oil, other conjugated isomers of linoleic acid, such as α-eleostearic acid (6.04%), catalpic acid (4.79%), and β-eleostearic acid (1.41%), were found in addition to punicic acid. In the work completed by Drinić et al. [31], a total of 18 fatty acids were identified in fresh pomegranate seed oil. SFAs accounted for 5.08–5.38% of all fatty acids, while MUFAs ranged from 6.44–6.81%. PUFAs, including punicic acid, were the main fatty acid class and accounted for 87.81–88.48% of all fatty acids. Punicic acid was the dominant fatty acid in all samples, and its content ranged from 36.06–70.58%. The content of oleic and linoleic acids was similar in all samples, ranging from 5.61 to 6.18% and from 5.00 to 5.54%, respectively. Studies by other researchers confirmed that the punicic acid content in different oils can varies significantly. The content of oil and specific fatty acids in pomegranate seeds is influenced by cultivation sites, harvest time, fruit genotypes, variety, and climatic conditions [9]. Changes in the content of punicic acid during storage may be related to the oxidation process occurring in this oil, since acids such as CLnA contain double bonds that are very sensitive to this process.

The specified health indices of atherogenicity, thrombogenicity, and hypocholesterolemia/hypercholesterolemia ratio provide information on the impact of fatty acids present in oils on human health in terms of the risk of atherosclerosis and the likelihood of thrombosis and atheroma. The oils recommended for consumption have a low atherogenicity index (AI < 1.0) and thrombogenicity index (TI < 0.5), and a high hypocholesterolemia/hypercholesterolemia (h/H) index. Consumption of products with lower AI is associated with a reduction in total cholesterol and low-density lipoprotein (LDL) cholesterol in human plasma [40], whereas consumption of products with a lower TI and higher h/H ratio may be beneficial in the prevention of cardiovascular heart disease [41]. The results showed that the one-month storage period did not significantly change the values of the health indicators of the tested oils. The oils were characterized by a low atherogenicity index (AI = 0.15, for oils A, C, and D) and a low thrombogenicity index (TI = 0.06 for oils A, C, and D), with the exception being oil B, for which the indexes were slightly higher: AI = 0.45 and TI = 0.19). Oil A was characterized by the highest value of the h/H index (13.01), while oil B had the lowest value (4.44). Summarizing the obtained results, it can be stated that the tested oils, like strawberry seed oil, blackcurrant seed oil, and cranberry seed oil, were characterized by beneficial nutritional and health values [42].

### 3.3. Fatty Acid Composition in sn-2 and sn-1,3 Positions of Triacylglycerols

**Figure 1:**
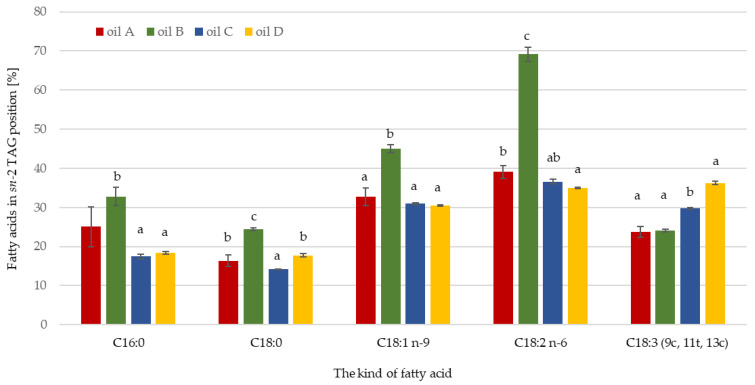
The content of the main fatty acids share in the *sn*-2 position of the triacylglycerols of the tested pomegranate seed oils. The different lowercase letters indicate significantly different values (*p* ≤ 0.05). Data are presented as mean values followed by standard deviation (±SD).

Triacylglycerols (TAGs) are the main components of vegetable oils. They consist of a glycerol skeleton combined with three esterified fatty acids. Not only the fatty acid profile but also the structure of TAGs significantly affect lipid metabolism in the human body. If SFAs are esterified in *sn*-1,3 positions, they are less well digested by the human body than unsaturated fatty acids in these positions. During digestion, pancreatic lipase releases saturated acids (C16:0; C18:0) from the outer positions, which tend to form insoluble and less well-absorbed calcium salts. The same acids esterified in the internal positions do not form calcium salts, and their absorption by the body is much better. The physical properties of fats such as melting point, solid fat content, crystal structure, and rate of oxidation and polymerization are also affected by the structure of TAGs [43,44,45]. Table 3 shows the fatty acid composition of the outer and inner TAG positions of the tested pomegranate seed oils. Figure 1 shows the content of the main fatty acids share in the *sn*-2 position of TAG.

Pomegranate seed oil A contained the most linoleic acid (32.24%) in the internal position, and its share in the *sn*-2 position of TAGs was more than 39%. Stearic acid was present in the least amount in the *sn*-2 position at 1.5%, and its share in the *sn*-2 position was 16.39%. The share of linoleic acid in the *sn*-2 position was over 33%, meaning its distribution was mainly in the internal positions of TAGs. The share of oleic acid in the *sn*-2 position was 32.75%, indicating an even distribution of this acid in the inner and outer positions. The other fatty acids in the TAGs were mainly distributed in the external positions, as their share in the *sn*-2 position was less than 33%. In oil B, punicic acid was present in the *sn*-2 position in the highest amount at 48.67%, but its share in this position was only 24.08%. On the contrary, stearic acid was present in the *sn-2* position (oil B) in the lowest amount at 2.10%, having also a low share in this position. The share of each of these two fatty acids in *sn*-2 below 33% indicated that they mainly occupied the outer positions in TAGs. Oleic and linolenic acids accounted for the largest share in the *sn*-2 position, at 45.01% and 69.17% respectively, indicating their distribution mostly in the internal position of TAGs. The share of palmitic acid in the *sn*-2 position of TAGs was 32.81%, which indicated an even distribution of this fatty acid in all the positions. The dominant acid in oil C in the inner TAG position was also punicic acid, the amount of which was 35.49%, while its share in this position was 29.71%, suggesting its slightly higher location in the outer TAG positions. In contrast, linolenic acid accounted for the highest share in the *sn*-2 position, at 36.59%; hence, this fatty acid was mostly present in the internal position of TAGs. The amount of stearic acid in the *sn*-2 position was 1.32% and its share in this position was 14.15%, meaning its distribution was mainly in the external positions of TAGs. Pomegranate seed oil D contained the highest amount of linoleic acid in the *sn*-2 position of TAGs, at 33.09%, with its share in this position being 34.98%. Linoleic acid and punicic acid in this oil occurred mainly in the internal position, as their share in this position was over 33%. The amount of stearic acid in the *sn*-2 position was 1.42%, while its share in this position was 17.73%, meaning its location was mostly in the external TAG positions of this oil.

There exists scant information in the available literature regarding the composition of fatty acids in the external and internal TAG positions of pomegranate seed oil. Therefore, it was necessary to examine the results obtained for other oils. In the study of Guan et al. [46], the composition of fatty acids in the TAGs of rapeseed oils was analyzed, with a high content of oleic acid and a low or high content of erucic acid found. The results showed that the oleic acid content in the high-oleic-acid rapeseed oil was about 80%, while in the low-erucic-acid oil the oleic acid was about 63%. The oleic acid in these oils was mainly distributed in the *sn*-2 position of TAGs. Rapeseed oil with a high-erucic-acid content (approx. 30%) contained only 24% of oleic acid in the middle TAG position. In this oil, erucic acid, with its long carbon chains, and oleic acid occurred mainly in the *sn*-1 and *sn*-3 positions. Vegetable oils contained mainly unsaturated fatty acids in the middle position (*sn*-2) and saturated acids in the outer positions (*sn*-1 and *sn*-3) [44].

### 3.4. Oxidation Induction Time (OIT)

The oxidation of oils reduces their quality, reduces their nutritional value, and produces an unpleasant taste and smell. Unfavorable changes in oils may already occur in oil seeds, but they are inevitable during oil production. The following factors are important: the degree of maturity, mechanical damage, and seed-processing conditions, such as pre-moistening, heating, and cold-pressing of the seeds. Pressure, pressing time, and temperature, as well as the sedimentation and filtration temperature of the oil, are also important [47,48]. Oxidative stability is one of the most important quality indicators of edible oils and fats. It is defined as the time between the sample reaching the measurement temperature and the rapid increase in peroxide production. In a thermoanalytical determination using the PDSC test, the oxidation induction times (determined based on the maximum rate of oxidation = maximum rate of heat flow) of four pomegranate seed oils and changes in the oxidation rate that occurred after a month of storage were determined. The results are presented in Table 4.

Immediately after opening, the longest oxidation induction time was for oil C, at 2.24 min, and the shortest was for oil D, at 0.52 min. After a month of storage, the oxidative stability of each tested oil decreased significantly. The longest induction time was observed for oil A (1.69 min) and the shortest for oil D (0.19 min). The highest drop (67%) in oxidative stability between the beginning and end of storage was observed for oil B after opening. On contrary, the lowest drop (22%) occurred for oil A. In the research presented by Siraj et al. [49], the antioxidant effect of pomegranate seed oil in various concentrations (5 and 7%) on the oxidation of rapeseed and sunflower oils during storage was assessed in comparison with the synthetic antioxidant butylated hydroxyanisole (BHA) using the furnace method defined by the Rancimat test and the Schaal oven storage stability test. The test results showed that pomegranate seed oil effectively delayed oxidation in vegetable oils during storage. Blends of the tested oils with the addition of 7% pomegranate seed oil showed higher induction time values, better antioxidant capacity, lower peroxide value, and slower degradation of tocopherols during storage. The results of this study suggest that pomegranate seed oil can be used as a potential antioxidant and an alternative to synthetic antioxidants.

It should be underlined that the oxidation induction times obtained by the Rancimat test are always higher than the OIT obtained by the PDSC method. PDSC analysis is carried out at elevated pressure (1400 kPa) and with pure oxygen flowing as a gas, while the Rancimat method uses air (*21% O_2_) and ambient pressure. In addition, the higher surface-to-volume ratio of the PDSC oil sample also plays an important role, leading to shorter analysis time [50,51]. According to Sharma et al. [20], increasing the oxygen pressure increases the local oxygen concentration as well as the oxygen diffusion rate as the number of moles of oxygen per unit volume increases. As a result, the OIT determined in a pure oxygen environment is usually lower than that obtained in air, provided that other experimental conditions remain the same. Overall, the lower oxidation induction time in the PDSC method is due to the small sample size and the use of pure oxygen, which makes oxygen readily available, but also to the use of high pressure, which speeds up oxidation [52].

The oxidative stability determined by the PDSC method is also correlated with the PUFA content: The higher the PUFA content, the shorter the oxidation induction time. Pomegranate seed oil is rich in PUFAs, which may explain its low oxidative stability. Additionally, punicic acid in oil B was located in the external position of TAGs, which is in accordance with the literature reports indicating that PUFAs located in the *sn*-1,3 TAG position reduce the oxidative stability of oils [53]. 

The oxidative stability of pomegranate seed oil is also affected by the content of the CLnA isomer of punicic acid. Many authors have reported that the presence of CLnA may contribute to the oxidative degradation of vegetable oils, thus affecting their stability [54,55]. A study by Yoshime et al. [56] also showed that the induction periods of the tested oils were shorter than those of other edible oils due to the high concentration of punicic acid. Therefore, pomegranate seed oil should be stored in a dry, cool, and dark place in sealed, dark glass bottles away from light. Factors such as temperature, light, and oxygen concentration have the greatest impact on the oxidative stability of oils.

Moreover, the presence of phenolic compounds in the oil is particularly important for the oxidative stability of the oil’s polyunsaturated fatty acids. Pomegranate seed oil is a rich source of compounds with antioxidant activity, mainly tocopherols [9]. The available literature [57,58] suggests that a high concentration of some phenolic-type antioxidants (e.g., BHA or tocopherols) can reverse their action and act as pro-oxidants. However, the influence of pomegranate seed oil on oxidative stability requires further research, which should include determining the content of antioxidant compounds and their total antioxidant activity.

### 3.5. Melting Characteristics

One of the main applications of DSC is the assessment of the physical properties of fats and oils. This method is used, among other things, to verify the quality of fats and detect adulteration. DSC is a method that is becoming more and more popular because it is fast and does not require any pre-treatment of the fat being tested nor any solvents harmful to the environment [59,60].

**Figure 2:**
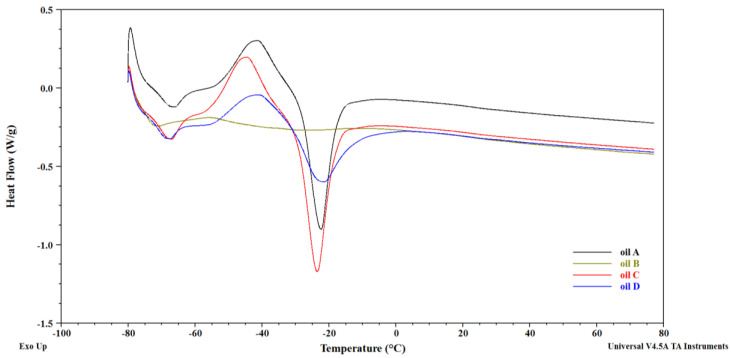
The representative melting curves of the tested pomegranate seed oils.

Melting and crystallization are two physical transformations used to characterize the thermal behavior of lipids. The melting and crystallization temperature of lipids decreases as the chain length decreases and the fatty acid degree of unsaturation increases. The different composition of TAGs influences the melting and crystallization curves of the tested lipids [59,60,61]. The measurement results are presented in the form of DSC curves, showing the amount of heat exchanged by the sample per unit of time as a function of temperature. An endothermic peak occurs when the temperature of the test sample is lower than the reference sample temperature, and an exothermic peak appears when the temperature of the test sample is higher than the reference sample temperature. The main exothermic peak in the cooling curves is related to the solidification of TAGs. However, endothermic peaks are related to the melting of oil [60,61]. Using the DSC method, the melting curves of four pomegranate seed oils were determined and presented in Figure 2. The characteristic temperatures connected to the onset, maximum, and end of the endothermic peaks are presented in Table 5. 

In Figure 2, for the all the tested oils, two endothermic peaks appear. The first peak on all DSC curves, with the maxima around −67 °C (oils A, C) and −72 °C (oil B), corresponded to the melting of the TAG fraction, where PUFAs, including punicic acid, pre-dominated. The second peak, with the maxima around −23 °C (oils A, D) and −31 °C (oil B), corresponded to the melting of the TAG fraction with MUFA domination. The shape of the first peak for oils A, C, and D resulted from the superposition of several TAG fractions with similar melting temperatures. This is a consequence of the known phenomenon of the polymorphism of natural oils and fats [37]. Probably, the dissimilar shape of the melting curve for oil B resulted from the different composition of fatty acid compared to other oils. Oil B was characterized by a significantly higher share of punicic acid and a lower share of α-linolenic acid. 

Khoddami et al. [54] conducted research on the thermal behavior of three pomegranate oils from Iran and Turkey. They concluded that the melting point range of all tested oils was between −69 °C and −13 °C. The melting curves of pomegranate oil samples were characterized, as in this study, by the presence of two main peaks. The minimum temperatures of the peaks were as follows: for the first oil, −68 °C and −17 °C; for the second one, −68 °C and −15 °C; and for the third one, −69 °C and −13 °C. In the DSC curves obtained by Teh and Birch (2013) [62] for oil from various seeds, the minima of the obtained thermal peaks were located in a slightly lower temperature range, i.e., for hemp seed oil, at −40 °C and −18 °C; for linseed oil, at −36 °C and −15 °C; and for rapeseed oil, at −23 °C and −9 °C. The lower temperature ranges in which endothermic peaks occur in pomegranate seed oils may result from the presence of a higher number of double bonds in the fatty acids of pomegranate oils than in the fatty acids of hemp, flax, and rapeseed oils. The chemical composition of the oil being tested has a significant impact on the thermal phenomena observed during cooling or heating. Different groups of TAGs influence the course of melting curves, which are unique to each fat [63].

## 4. Conclusions

Differences in the fatty acid composition as well as different hydrolytic and oxidative stability before and after storage were observed in the four tested commercial pomegranate seed oils. The results show that most of the tested oils had a low content of primary oxidation products immediately after opening, while storage significantly increased their content. Moreover, the chromoatographic analysis indicated that only commercial oil B had a fatty acid composition comparable to the fatty acid profile of pure pomegranate seed oil. It turns out that not all commercial oils have as high a content of punicic acid as natural pomegranate seed oil. Nevertheless, taking into account the results regarding health indicators, it can be concluded that all tested pomegranate seed oils were characterized by beneficial nutritional and health values.

Due to the high content of punicic acid, the oxidative stability of pomegranate seed oil was low. This is a serious problem because commercial oils should retain their properties throughout their shelf life. Reducing oxidation-promoting factors or supplementing products with antioxidants can lead to products with an extended shelf life. Different fatty acid compositions and DSC melting curves may indicate that some commercial pomegranate seed oils are blended with cheaper and/or lower quality oils. However, this area of research requires more attention and further research.

## Figures and Tables

**Table 1:** Average acid and peroxide values of tested oils immediately after opening and after one month of storage.

Oil	Storage (Month)	Acid Value (mg KOH/g Oil)	Peroxide Value (meq O_2_ /kg Oil)
**A**	0	1.97 ^a^ ± 0.01	10.92 ^b^ ± 0.61
	1	2.01 ^a^ ± 0.05	35.5 ^f^ ± 0.06
**B**	0	2.72 ^b^ ± 0.03	6.34 ^a^ ± 0.05
	1	2.82 ^b^ ± 0.03	18.2 ^c^ ± 0.21
**C**	0	1.76 ^a^ ± 0.02	12.08 ^b^ ± 0.3
	1	1.88 ^a^ ± 0.03	37.42 ^g^ ± 0.38
**D**	0	6.12 ^c^ ± 0.20	19.31 ^d^ ± 0.23
	1	6.25 ^c^ ± 0.10	24.85 ^c^ ± 0.17

Determined data are presented as mean values followed by standard deviation (±SD). The different lowercase letters indicate significantly different values (*p* ≤ 0.05).

**Table 2:** Fatty acid composition (%) and health indices for tested pomegranate seed oils.

	Composition after Opening (%)				Composition after Storage (%)			
Fatty Acid	Oil A	Oil B	Oil C	Oil D	Oil A	Oil B	Oil C	Oil D
**C16:0**	4.28 ± 0.04	3.81 ± 0.00	4.50 ± 0.01	5.02 ± 0.06	4.22 ± 0.02	3.21 ± 0.01	4.34 ± 0.02	4.52 ± 0.40
**C18:0**	3.05 ± 0.01	2.87 ± 0.13	3.11 ± 0.01	2.67 ± 0.01	2.98 ± 0.03	2.64 ± 0.01	3.09 ± 0.01	2.68 ± 0.04
**C18:1** **n-9**	18.92 ± 0.06	7.65 ± 0.53	19.95 ± 0.08	17.35 ± 0.06	18.74 ± 0.06	6.91 ± 0.06	19.63 ± 0.02	17.30 ± 0.34
**C18:2** **n-6**	27.47 ± 0.06	8.40 ± 1.17	29.01 ± 0.09	31.53 ± 0.17	27.34 ± 0.05	7.15 ± 0.01	28.62 ± 0.02	30.67 ± 0.71
**C18:3** **n-3**	0.15 ± 0.01	0.02 ± 0.01	0.04 ± 0.01	0.81 ± 0.01	0.15 ± 0.01	0.02 ± 0.01	0.04 ± 0.01	0.78 ± 0.08
**C20:0**	0.47 ± 0.01	0.57 ± 0.06	0.44 ± 0.01	0.43 ± 0.01	0.46 ±0.01	0.56 ± 0.00	0.45 ± 0.01	0.60 ± 0.21
**C20:1** **n-9**	0.61 ± 0.01	0.81 ± 0.06	0.56 ± 0.01	0.59 ± 0.01	0.59 ± 0.01	0.87 ± 0.01	0.56 ± 0.01	0.70 ± 0.12
**C20:3** **n-3**	0.49 ± 0.01	0.10 ± 0.05	0.42 ± 0.02	0.35 ± 0.05	0.44 ± 0.03	0.13 ± 0.01	0.45 ± 0.01	0.35 ± 0.16
**C18:3** **(9c, 11t, 13c)**	41.68 ± 0.15	67.36 ± 1.07	39.82 ± 0.16	27.03 ± 0.14	42.12 ± 0.34	69.74 ± 0.17	40.48 ± 0.11	27.41 ± 0.47
**Other 1**	2.51 ± 0.01	6.83 ± 0.43	1.85 ± 0.01	8.74 ± 0.06	2.56 ± 0.10	7.02 ± 0.25	2.04 ± 0.03	9.09 ± 0.03
**Other 2**	0.41 ± 0.01	1.61 ± 0.15	0.29 ± 0.01	5.51 ± 0.05	0.43 ± 0.02	1.78 ± 0.01	0.33 ± 0.01	7.59 ± 2.15
**Σ MUFA**	19.50 ^c^	8.45 ^a^	20.51 ^d^	17.94 ^b^	19.33 ^c^	7.77 ^a^	20.19 ^cd^	17.00 ^b^
Σ PUFA *	28.1 ^b^	8.51 ^a^	29.46 ^b^	32.67 ^c^	27.92 ^b^	7.29 ^a^	29.10 ^b^	31.80 ^c^
**Σ SFA**	7.8 ^cd^	7.25 ^b^	8.05 ^cd^	8.11 ^d^	7.66 ^c^	6.40 ^a^	7.87 ^cd^	7.80 ^cd^
**CLnA**	41.68 ^c^	67.36 ^d^	39.84 ^b^	27.03 ^a^	42.10 ^c^	69.74 ^e^	40.48 ^bc^	27.41 ^a^
**Σ other**	2.92	8.43	2.14	14.25	2.99	8.8	2.36	15.99
**Health Indices**								
**AI**	0.15	0.45	0.15	0.15	0.15	0.44	0.14	0.14
**TI**	0.06	0.19	0.06	0.06	0.07	0.20	0.07	0.06
**h/H**	13.01	4.44	13.00	12.95	13.13	4.50	13.31	13.99

MUFA—monounsaturated fatty acid; PUFA—polyunsaturated fatty acid; SFA—saturated fatty acid; CLnA—conjugated linolenic acid; AI—atherogenic index; TI—thrombogenic index; h/H—hypocholesterolemic/hypercholesteremic index. * Total PUFAs without CLnA acid. The different lowercase letters indicate significantly different values (*p* ≤ 0.05). Data are presented as mean values followed by standard deviation (±SD).

**Table 3:** Fatty acid composition of the outer (*sn*-1,3) and inner (*sn*-2) triacylglycerol (TAG) positions of the tested pomegranate seed oils.

Fatty Acid	Composition (%)				
		Oil A	Oil B	Oil C	Oil D
**C16:0**	TAG	4.28 ± 0.04 ^b^	3.81 ± 0.01 ^a^	4.50 ± 0.01 ^c^	5.02 ± 0.06 ^d^
	*sn*-2	3.22 ± 0.66 ^ab^	3.75 ± 0.26 ^b^	2.36 ± 0.08 ^a^	2.76 ± 0.05 ^ab^
	*sn*-1,3	4.81 ± 0.33 ^b^	3.84 ± 0.13 ^a^	5.57 ± 0.04 ^c^	6.15 ± 0.02 ^d^
**C18:0**	TAG	3.05 ± 0.01 ^bc^	2.87 ± 0.13 ^b^	3.11 ± 0.03 ^c^	2.67 ± 0.01 ^a^
	*sn*-2	1.5 ± 0.13 ^a^	2.10 ± 0.03 ^b^	1.32 ± 0.01 ^a^	1.42 ± 0.04 ^a^
	*sn*-1,3	3.83 ± 0.07 ^b^	3.26 ± 0.01 ^a^	4.01 ± 0.01 ^c^	3.30 ± 0.02 ^a^
**C18:1 n-9**	TAG	18.92 ± 0.06 ^c^	7.65 ± 0.53 ^a^	19.95 ± 0.08 ^d^	17.35 ± 0.06 ^b^
	*sn*-2	18.59 ± 1.29 ^c^	10.33 ± 0.25 ^a^	18.14 ± 0.15 ^c^	15.88 ± 0.08 ^b^
	*sn*-1,3	19.09 ± 0.65 ^c^	6.31 ± 0.13 ^a^	20.86 ± 0.07 ^d^	18.09 ± 0.04 ^b^
**C18:2 n-6**	TAG	27.47 ± 0.06 ^b^	8.40 ± 0.06 ^a^	29.01 ± 0.09 ^c^	31.53 ± 0.17 ^d^
	*sn*-2	32.24 ± 1.35 ^b^	17.43 ± 0.46 ^a^	31.84 ± 0.50 ^b^	33.09 ± 0.17 ^b^
	*sn*-1,3	25.09 ± 0.68 ^b^	3.89 ± 0.23 ^a^	27.60 ± 0.25 ^c^	30.75 ± 0.08 ^d^
**C18:3** **(9c, 11t, 13c)**	TAG	41.68 ± 0.15 ^c^	67.36 ± 1.07 ^d^	39.82 ± 0.16 ^b^	27.03 ± 0.14 ^a^
	*sn*-2	29.72 ± 1.66 ^a^	48.67 ± 0.54 ^c^	35.49 ± 0.34 ^b^	29.41 ± 0.30 ^a^
	*sn*-1,3	47.66 ± 0.83^c^	76.71 ± 0.27 ^d^	41.99 ± 0.17 ^b^	25.84 ± 0.15 ^a^

The different lowercase letters indicate significantly different values (*p* ≤ 0.05). Data are presented as mean values followed by standard deviation (±SD).

**Table 4:** Oxidation induction time (OIT) of tested pomegranate seed oils at 120 °C.

Oil	Storage (Month)	Oxidation Induction Time (Min)
**A**	0	2.16 ^e^ ± 0.04
	1	1.69 ^de^ ± 0.33
**B**	0	0.90 ^bc^ ± 0.01
	1	0.30 ^ab^ ± 0.09
**C**	0	2.24 ^e^ ± 0.15
	1	1.13 ^cd^ ± 0.17
**D**	0	0.52 ^abc^ ± 0.19
	1	0.19 ^a^ ± 0.02

The different lowercase letters indicate significantly different values (*p* ≤ 0.05). Data are presented as mean values followed by standard deviation (±SD).

**Table 5:** Characteristic temperatures corresponding to the onset (t_onset_), maximum (t_m_), and offset (t_offset_) of the peaks on the melting curves of the tested pomegranate seed oils.

Oil.	Peak 1			Peak 2		
	t_onset_ (°C)	t_m_ (°C)	t_offset_ (°C)	t_onset_ (°C)	t_m_ (°C)	t_offset_ (°C)
**A**	−79.94 ^a^ ± 0.05	−66.92 ^a^ ± 0.01	−42.30 ^a^ ± 0.31	−29.44 ^a^ ± 0.25	−22.80 ^a^ ± 0.43	−15.42 ^d^ ± 0.54
**B**	−79.80 ^a^ ± 0.13	−72.37 ^d^ ± 0.33	−57.75 ^d^ ± 0.19	−53.52 ^d^ ± 0.06	−30.65 ^c^ ± 0.40	−14.99 ^c^ ± 0.08
**C**	−79.94 ^a^ ± 0.07	−67.00 ^b^ ± 0.04	−46.55 ^c^ ± 0.01	−31.96 ^b^ ± 0.12	−23.72 ^b^ ± 0.01	−14.59 ^b^ ± 0.04
**D**	−79.92 ^a^ ± 0.04	−68.51 ^c^ ± 0.16	−44.68 ^b^ ± 0.06	−39.75 ^c^ ± 0.16	−22.77 ^a^ ± 0.22	−4.68 ^a^ ± 0.18

The different lowercase letters indicate significantly different values (*p* ≤ 0.05). Data are presented as mean values followed by standard deviation (±SD).

## Data Availability

The original contributions presented in the study are included in the article, further inquiries can be directed to the corresponding author.
